# Comparison of published core outcome sets with outcomes recommended in regulatory guidance from the US Food and Drug Administration and European Medicines Agency: cross sectional analysis

**DOI:** 10.1136/bmjmed-2022-000233

**Published:** 2022-11-03

**Authors:** Ian J Saldanha, Susanna Dodd, Rebecca Fish, Sarah L Gorst, Deborah A Hall, Pamela Jacobsen, Jamie J Kirkham, Dominic Trepel, Paula R Williamson

**Affiliations:** 1 Department of Health Services, Policy, and Practice, Brown University School of Public Health, Providence, RI, USA; 2 Department of Health Data Science, University of Liverpool, Liverpool, UK; 3 Faculty of Biology, Medicine, and Health, University of Manchester, Manchester, UK; 4 Department of Psychology, Heriot-Watt University - Malaysia Campus, Putrajaya, Wilayah Persekutuan Putrajaya, Malaysia; 5 Department of Psychology, University of Bath, Bath, UK; 6 Trinity Institute of Neurosciences, Trinity College Dublin, Dublin, Ireland; 7 Global Brain Health Institute, University of California San Francisco, San Francisco, CA, USA

**Keywords:** Internal medicine, Epidemiology

## Abstract

**Objective:**

To compare the outcomes in published core outcome sets with the outcomes recommended in corresponding guidance documents from the European Medicines Agency (EMA) and US Food and Drug Administration (FDA), matched by health condition.

**Design:**

Cross sectional analysis.

**Setting:**

US and Europe.

**Population:**

Sample of core outcome sets related to drugs, devices, and gene therapy that involved patients in the consensus process, published between 1 January 2015 and 31 December 2019; and corresponding EMA and FDA guidance documents.

**Main outcome measures:**

The extent of matches between outcomes included within core outcome sets and those recommended in corresponding EMA and FDA guidance documents were assessed. Matches were considered to be general (ie, non-specific) or specific (ie, exact). General matches were assessed to determine whether the core outcome set or guidance document outcome was narrower.

**Results:**

Relevant guidance documents were found for for 38 (39%) of 98 eligible published core outcome sets. Among outcomes in core outcome sets, medians of 70% (interquartile range 48-86%) and 52% (33-77%) were matches with outcomes recommended in EMA and FDA documents, respectively. Medians of 46% (27-68%) and 26% (18-46%) were specific matches with outcomes in EMA and FDA documents, respectively. When outcomes were generally matched, the outcomes from core outcome sets were more frequently narrower than the regulatory outcomes (83% and 75% for EMA and FDA, respectively).

**Conclusion:**

Greater adoption of, and reference to, core outcome sets in regulatory guidance documents can encourage clinical trialists, especially those in industry, to measure and report consistent and agreed outcomes and improve the quality of guidance. Given the overlap between outcomes in core outcome sets and regulatory guidance, and given that most core outcome sets now involve patients in the consensus process, these sets could serve as a useful resource for regulators when recommending outcomes for studies evaluating regulated products. Developers are encouraged to appraise recommended outcomes in salient regulatory documents when planning a core outcome set.

**WHAT IS ALREADY KNOWN ON THIS TOPIC**

- Core outcome sets are agreed standardised sets of outcomes within specific clinical topic areas
- Clinical trialists are highly influenced by regulators of drug and device products

**WHAT THIS STUDY ADDS**

- The extent of matches was assessed between outcomes included in published core outcome sets and those recommended in corresponding guidance documents from the European Medicines Agency (EMA) and US Food and Drug Administration (FDA)
- Among outcomes in published sets, medians of 70% (interquartile range 48-86%) and 52% (33-77%) were matches with outcomes in EMA and FDA documents, respectively

**HOW THIS STUDY MIGHT AFFECT RESEARCH, PRACTICE, OR POLICY**

- Given the overlap between outcomes in core outcome sets and regulatory guidance and the increasing patient involvement in the generation of most core outcome sets, these sets could be a useful resource for regulators when recommending outcomes for studies evaluating regulated products
- Developers should appraise recommended outcomes in salient regulatory documents when planning a core outcome set

## Introduction

Core outcome sets are agreed standardised sets of outcomes within specific clinical topic areas.^1^ These sets are developed to inform either research or clinical practice and are generally determined by an initial systematic review (to identify all potential outcomes), followed by a process to prioritise the most important outcomes based on consensus among health professionals, researchers, policymakers, and patients or their representatives.^2^ The Core Outcome Measures in Effectiveness Trials (COMET) Initiative brings together individuals and groups working on developing and applying core outcome sets and improving their development process.^3 4^ COMET maintains a free, publicly available, searchable database of completed and ongoing development projects of core outcome sets.^3^

Core outcome sets are developed for two main reasons. They help ensure that the priorities and expertise of key stakeholders inform the recommended set of outcomes to measure in clinical trials for a given health condition, and that the results of those trials, having reported at least the core outcomes in common, can be incorporated into systematic reviews and meta-analyses to inform regulatory and healthcare guidance and decision making.^5–11^

The use of core outcome sets is increasingly recommended by a broad set of stakeholders in the evidence ecosystem, including trial funders and those who use the results of trials (eg, policymakers).^12^ Several trial funders, such as the UK National Institute for Health and Care Research (NIHR), the US Patient-Centred Outcomes Research Institute, the Irish Health Research Board, and the Netherlands Organisation for Health Research and Development, recommend that applicants for trial funding consider using a core outcome set if one exists.^13^ For example, the NIHR refers applicants to the COMET Database, suggesting that established core outcomes be included "unless there is good reason to do otherwise."^14^ The SPIRIT reporting guidelines for clinical trial protocols recommend that trial authors consult the COMET Database to identify relevant core outcome sets when choosing outcomes for the trial.^15 16^ Organisations that rely on evidence to support improvement in healthcare services (eg, the Healthcare Quality Improvement Partnership (HQIP)^17^) and to inform decision making (eg, the UK National Institute for Health and Care Excellence (NICE)^18^) are also recognising the relevance of core outcome sets in their work. The HQIP tool describing key features of national clinical audits and registries states that the rationale for quality improvement objectives should consider relevant outcomes from the COMET Database.^17^ In 2018, NICE guidance on methods to determine relevant guideline outcomes was updated to indicate that core outcome sets should be used, if suitable based on their quality and validity.^18^

Notwithstanding the increase in endorsements of core outcome sets by various bodies, trialists are also highly influenced by regulators of drug and device products. Although regulatory guidelines are not legally binding documents, they are an important source of guidance for trialists. Two of the world’s prominent healthcare regulators are the European Medicines Agency (EMA) and the US Food and Drug Administration (FDA). The EMA publishes scientific guidelines to inform marketing authorisation applications for human medicines.^19^ Similarly, the FDA publishes official Guidance Documents and other documents covering various classes of regulated products, such as biological medicines, drugs, medical devices, and food.^20^ The FDA also publishes general guidance on study design and outcomes, such as guidance on the conduct of randomised trials during the covid-19 pandemic^21^ and on the use of patient-reported outcome measures.^22^ Guidance from the EMA and FDA is highly influential on what research is commissioned, particularly where evidence gaps are a source of uncertainty for existing guidance.

Core outcome sets are developed using methods that incorporate patient and clinician (and other stakeholder) preferences for outcomes. Therefore, if suggested outcomes in regulatory guidance documents align with core outcome sets, such alignment would bring these salient outcomes to the attention of investigators as they design their clinical trials. As a first step, we need a better understanding of the similarities and differences between the outcomes included in core outcome sets and the outcomes recommended by regulatory bodies. In the present study, we aimed to conduct a systematic assessment of the degree of concordance between outcomes in published outcome sets and outcomes recommended in FDA and EMA guidance documents, matched by health condition.

## Methods

We published the protocol for this study prospectively.^23^

### Selection of core outcome sets

We examined all core outcome sets for research (including those intended for both research and practice) that involved patients in the consensus process and were published between 1 January 2015 and 31 December 2019. Selection of only those core outcome sets published in the past five years (at the time of beginning our study) that involved patients probably increased the number of COS-STAD standards (Core Outcome Set Standards for Development^2^) that are met by this sample of core outcome sets. To maximise relevance to regulatory guidance, we restricted the study sample to those sets relating to any intervention or specifically to drug, device, or gene therapy interventions; we excluded sets relating exclusively to surgical interventions because such procedures are not subject to EMA or FDA regulatory oversight.

### Identification of matching regulatory guidance documents

For each eligible core outcome set, we identified EMA/FDA guidance documents that addressed similar health conditions, published up to April 2021. Because we are not aware of a readily searchable electronic database for guidance documents, we searched websites of the EMA^19^ and FDA,^20^ using the key clinical terms and synonyms as search terms. We refined these searches using Google’s site specific search capability. For example, if searching for guidance documents relating to diabetes, we searched for "diabetes site: fda.gov" on the FDA website and for "diabetes site: ema.europa.eu" on the EMA website. We considered addendums to guidance documents as separate guidance documents because they usually refer to different target populations and/or interventions from those in the original documents. A core outcome set could be matched to more than one guidance document, and vice versa. For all core outcome sets, two investigators (from among IJS, SD, RF, SLG, DAH, PJ, JJK, and DT, each of whom is experienced in core outcome set development or methodology) independently ran searches for guidance documents and resolved disagreements in search results through discussion, consulting SD and PRW as needed.

### Assessing overlap in scope between core outcome sets and matched guidance documents

**Figure 1:**
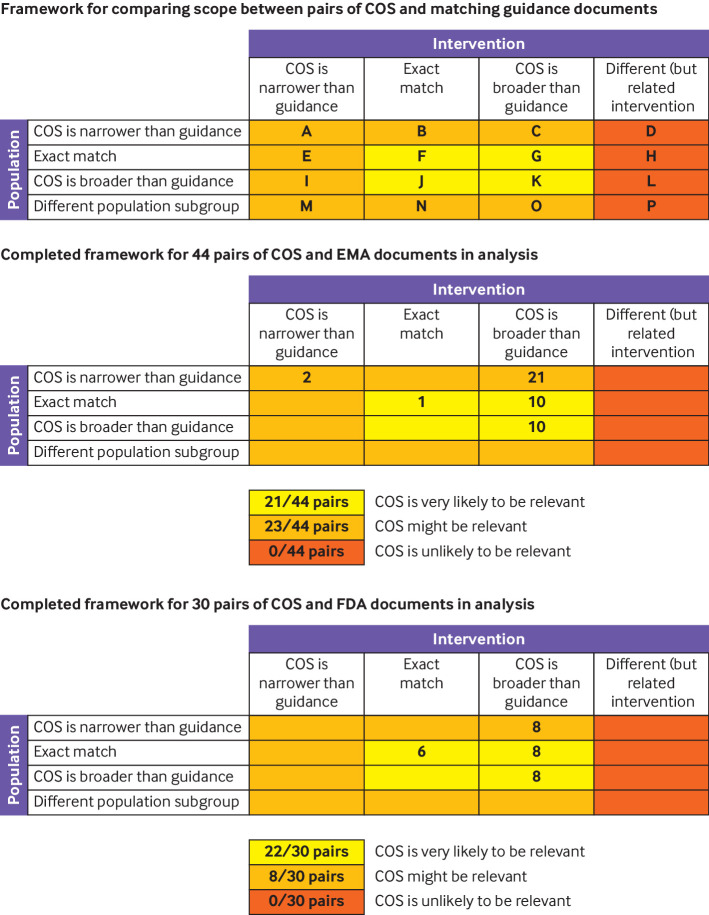
Comparison frameworks of core outcome sets (COS) matched with guidance documents from the EMA (European Medicines Agency) and FDA (US Food and Drug Administration) in the present analysis. Top panel shows the framework for comparing scope between pairs of COS and matching guidance documents (adapted with permission^9^). Middle panel shows the completed framework for 44 pairs of COS and EMA regulatory guidance documents in the present analysis. Bottom panel shows the completed framework for 30 pairs of COS and FDA regulatory guidance documents in the present analysis

We considered the scope of a given core outcome set as the reference and any given regulatory guidance document to match if they were at least generally matched in terms of both clinical condition/disease and intervention. We used a previously developed framework by Saldanha et al to assess the overlap (figure 1, top panel).^9^ We considered core outcome sets and regulatory documents to be matched only in scenarios represented by scenarios A-C, E-G, or I-K (ie, those corresponding to at least a general match in both intervention and population between the core outcome set and guidance document; figure 1, top panel). Table 1 provides examples of these scenarios.

**Table 1:** Examples of overlap in scope between core outcome sets and matched guidance documents

Scenario*	Population	Intervention	Example title of COS	Example title of guidance document	Regulator
A	COS is narrower	COS is narrower	How to evaluate the clinical outcome of joint-preserving treatment for osteonecrosis of the femoral head: development of a core outcome set	Guideline on clinical investigation of medicinal products used in the treatment of osteoarthritis	EMA
B	COS is narrower	Exact match	None	None	N/A
C	COS is narrower	COS is broader	Selecting Core Outcomes for Randomised Effectiveness trials In Type 2 diabetes (SCORE-IT): a patient and healthcare professional consensus on a core outcome set for type two diabetes	Guideline on clinical investigation of medicinal products in the treatment or prevention of diabetes mellitus	EMA
E	Exact match	COS is narrower	None	None	N/A
F	Exact match	Exact match	Achieving consensus on minimum data items (including core outcome domains) for a longitudinal observational cohort study in rheumatoid arthritis	Clinical development programmes for drugs, devices, and biological products for the treatment of rheumatoid arthritis	FDA
G	Exact match	COS is broader	Toward establishing core outcome domains for trials in kidney transplantation: report of the standardised outcomes in nephrology-kidney transplantation consensus workshops	Delayed graft function in kidney transplantation: developing drugs for prevention	FDA
I	COS is broader	COS is narrower	None	None	N/A
J	COS is broader	Exact match	None	None	N/A
K	COS is broader	COS is broader	Chronic rhinosinusitis outcome Measures (CHROME) – developing a core outcome set for trials of interventions in chronic rhinosinusitis	Non-allergic rhinitis: developing drug products for treatment	FDA

COS=core outcome set; EMA=European Medicines Agency; FDA=US Food and Drug Administration; N/A=not applicable.

*Letters relate to those in figure 1, top panel.

### Extracting information

For each core outcome set, we used an existing database of previously extracted information regarding recommended core outcomes. This database contains all published core outcome sets and is compiled using data extracted for an annually updated systematic review of published core outcome sets.^7 24–27^ We also reviewed the articles describing each core outcome set (also obtained from the COMET Database) to confirm the list of core outcomes. In the one instance where an article recommended additional outcomes but specified that these were not part of the core outcome set (adverse events in a core outcome set for haemophilia),^28^ we included those outcomes when comparing the core outcome set to the regulatory document because those outcomes were recommended. We also assessed whether the core outcome set referred to the corresponding regulatory document.

To facilitate standardised data extraction across the team, all investigators participated in an initial pilot exercise using two pairs of core outcome sets and regulatory documents. After that, for the remaining data extraction, two investigators (from among IJS, SD, RF, SLG, DAH, PJ, JJK, and DT) independently extracted all outcomes from each pair of core outcome set and regulatory guidance document. We extracted outcomes regardless of the outcome’s location in the document or the outcome’s status as primary, secondary, or other. Disagreements between pairs of extractors were resolved by discussion among the pairs (and, when needed, with SD).

### Matching of outcomes between core outcome set and relevant guidance documents

We focused on the outcome domains (eg, pain for the "what" of an outcome). We did not examine whether the "how" of an outcome (eg, one pain measurement instrument versus another) matched. We considered matching of outcomes separately for each pair of core outcome set and guidance document.

Consistent with previous work,^9 29^ we considered an outcome in a core outcome set and an outcome in a guidance document to be matched if they were generally or specifically related. Outcome pairs were thus matched generally if one document specified a broad outcome (eg, "disease activity"), while the other was more explicit (eg, "joint damage"); or matched specifically if both documents specified the same explicit outcome (eg, "overall survival" and "all-cause mortality").

Measurement instruments (eg, multi-component questionnaires on quality of life) recommended in guidance documents deserve special mention. If the outcome instrument recommended in a guidance document overlapped with an outcome in a core outcome set, we considered the outcomes as generally matched if the guidance outcome included an overall summary measure that covered more domains (eg, health related quality of life) than just the core outcome set's outcome (eg, physical functioning); or as specifically matched if the instrument covered only the core outcome set's outcome. For all generally matched pairs of outcomes, we also assessed which of the two outcomes was broader.

### Statistical analysis

We calculated descriptive statistics (percentages and medians with interquartile ranges) for core outcome sets and relevant guidance documents. We calculated the median percentages of outcomes in core outcome sets that were specific matches, general matches, and non-matches with outcomes in relevant guidance documents overall as well as separately for the EMA and FDA. We constructed scatter plots and used kernel (non-parametric) smoothing to depict potential relations between percentage matching (ie, percentage of outcomes in the core outcome sets that were matched to outcomes in the guidance documents) and the number of outcomes in the core outcome sets. For the kernel smoothing, we used the rule-of-thumb method to calculate window sizes. We conducted all analyses using Stata version 16 (College Station, TX). We estimated the medians (and interquartile ranges) of the proportion of outcomes that overlapped between the core outcome sets and guidance documents.

### Patient and public involvement

Patients and the public were not involved in the design, conduct, reporting, or dissemination plans of this research, as this was a methodological study.

## Results

### Included core outcome sets

**Figure 2:**
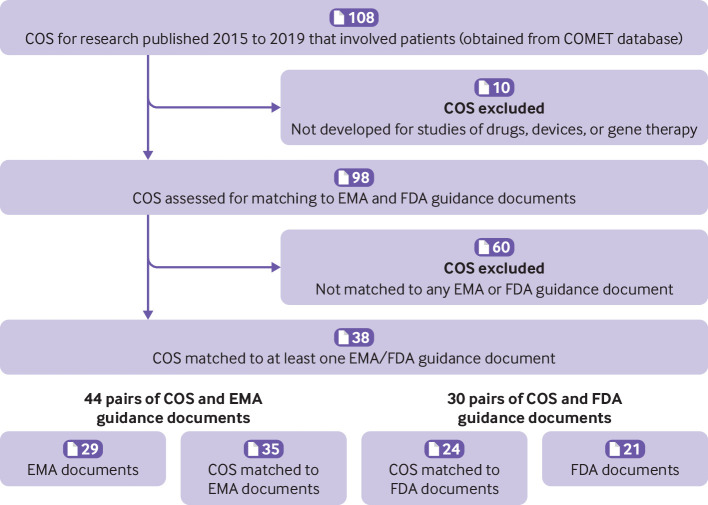
Study flow of COS (core outcome sets) and regulatory guidance documents from the EMA and FDA (European Medicines Agency) and FDA (US Food and Drug Administration). COMET=core outcome measures for effectiveness trials. One COS could be relevant to multiple regulatory guidance documents and vice versa

Based on a search of the COMET Database, a total of 108 core outcome sets for research, with patients included in the consensus process, were published between 1 January 2015 and 31 December 2019 (figure 2). We excluded 10 core outcome sets relating to specific interventions other than drugs, devices, or gene therapy.

Among the remaining 98 core outcome sets, we identified at least one regulatory guidance document for 38 (39%) sets. Table 2 summarises these 38 core outcome sets. Just under a third (32%) of these 38 sets were published in 2017. The most frequent topic areas were neurology (18%), cancer (13%), gastroenterology (13%), and pain (11%). Most (82%) core outcome sets were not developed for a specific type of intervention (ie, they were developed for any intervention). The 38 core outcome sets included a median of eight outcomes (interquartile range 6-11) per set. None of the core outcome sets referred to their corresponding regulatory documents.

**Table 2:** Characteristics of core outcome sets included in analysis

Characteristic	No (%) of core outcome sets (n=38)
**Year of publication**	
2015	6 (16)
2016	3 (8)
2017	12 (32)
2018	9 (24)
2019	8 (21)
**Topic area**	
Neurology	7 (18)
Cancer	5 (13)
Gastroenterology	5 (13)
Pain	4 (11)
Diabetes	3 (8)
Rheumatoid arthritis	2 (5)
Allergy or infections	2 (5)
Other	10 (26)
**Type of intervention targeted**	
Any	31 (82)
Drugs	4 (11)
Devices/surgeries	2 (5)
Gene therapy	1 (3)
**No of outcomes per core outcome set**	
Median	8
Interquartile range	6-11
Range	2-38
Mean	10.0
Standard deviation	4.1

### Overlap in timing and scope

We found 29 matching EMA guidance documents for 35 core outcome sets (figure 2). These EMA guidance documents were published between 2005 and 2021. Of the 44 pairs of core outcome sets matched with EMA documents, the core outcome set was published before the EMA document in 15 (34%) pairs, published in the same year in five (11%) pairs, and published after the EMA document in 24 (55%) pairs. Figure 1 (middle panel) shows these 44 pairs. The core outcome set was very likely to be relevant for 21 (48%) pairs and may have been relevant for 23 (52%) pairs. The most frequent was scenario C (figure 1, top panel; ie, the core outcome set's population was narrower than the population outlined in the EMA guidance document, but the core outcome set described a broader intervention or set of interventions than the EMA guidance document; 21 (48%) pairs). Both the population and the intervention in the core outcome set and the EMA guidance were exact in scope (ie, scenario F in figure 1 (top panel)) in only one (2.3%) pair.

We found 21 matching FDA guidance documents for 24 core outcome sets (figure 1). These FDA guidance documents were published between 1981 and 2020. Of the 30 pairs of core outcome sets matched with FDA documents, the core outcome set was published before the FDA document in 12 (40%) pairs, in the same year in four (13%) pairs, and after the FDA document in 14 (47%) pairs. Figure 1 (bottom panel) shows these 30 pairs. The core outcome set was very likely to be relevant for 22 (73%) pairs and may have been relevant for eight (27%) pairs. For one in five pairs (20%), both the population and the intervention were exact in scope (ie, scenario F in figure 1, top panel).

### Overlap in outcomes

For the 44 pairs of core outcome sets and EMA guidance documents, a median of 70% of outcomes in the core outcome sets (interquartile range 48-86%) were *s*pecific or general matches to outcomes in corresponding guidance documents (table 2). A median of 46% (27-68%) were specific matches. Among general matches in outcomes, the EMA outcome was broader in 83% and the COS outcome was broader in 17%. The scatter plot in online supplemental figure 1A suggests a generally inverse relation between the number of outcomes in the core outcome set and the percentage match between the outcomes in the core outcome set and the EMA guidance document. This relation was true for all matches (blue smoothed curve) as well as for specific matches in particular (green smoothed curve).

10.1136/bmjmed-2022-000233.supp1 Supplementary data

For the 30 pairs of core outcome sets and FDA guidance documents, a median of 52% of outcomes in the core outcome sets (interquartile range 33-77%) were specific or general matches to outcomes in corresponding guidance documents (table 3). A median of 26% (18-46%) were specific matches. Among general matches in outcomes, the FDA outcome was broader in 75% and the COS outcome was broader in 25%. The scatter plot in online supplemental figure 1B suggests a generally inverse relation between the number of outcomes in the core outcome set and the percentage match between the outcomes in the core outcome set and the FDA guidance document. This relation was true for all matches (blue smoothed curve) as well as for specific matches in particular (green smoothed curve).

**Table 3:** Median (interquartile range) percentage of overlap in outcomes between pairs of core outcome sets (COS) and matching guidance documents from the European Medicines Agency (EMA) and US Food and Drug Administration (FDA)

Pairs	Specific matches (%)	General matches (%)	Specific or general matches (%)
COS and EMA (n=44)	46 (27-68)	13 (0-27)	70 (48-86)
COS and FDA (n=30)	26 (18-46)	11 (0-37)	52 (33-77)

### Examples of matching

Table 4 provides examples of specific matches, general matches, and non-matches between pairs of outcomes in EMA/FDA guidelines and outcomes recommended in corresponding core outcome sets. For generally matched outcomes, table 4 also includes our assessment of whether the outcome was narrower, broader, or neither in the core outcomes sets than in the matched regulatory documents.

**Table 4:** Examples of specific matches, general matches, and non-matches between outcomes in regulatory guidance documents and corresponding relevant core outcome sets

Clinical area	Outcome in EMA/FDA guideline	Outcome in COS	Type of match	Comparative assessment of breadth of outcomes
Back pain	Pain intensity	Pain intensity	Specific	—
Back pain	Quality of life	Health related quality of life	Specific	—
Neurodisability	Sleep disturbance	Sleep	Specific	—
Neurodisability	Behavioural reactions	Behaviour	Specific	—
Back pain	Emotional functioning	Depression	General	Outcome in COS is narrower
Neurodisability	Activities of daily living	Toileting	General	Outcome in COS is narrower
Coronary artery disease	Symptomatic improvement	Angina	General	Outcome in COS is narrower
Type 1 diabetes	Quality of life	Diabetes related quality of life	General	Outcome in COS is narrower
Prostate cancer	Overall survival	Death from prostate cancer	General	Outcome in COS is narrower
Type 1 diabetes	Nocturnal hypoglycaemia	Hypoglycaemia	General	Outcome in COS is broader
Prostate cancer	Time to need of radical treatment	Treatment failure	General	Outcome in COS is broader
Rolandic epilepsy	Coordination	Gross motor function	General	Outcome in COS is broader
Multiple sclerosis	Relapse	Employment	Not a match	—
Rheumatology	Analgesic Use	Utility	Not a match	—
Type 1 diabetes	Body weight	Perceived level of control over diabetes	Not a match	—

COS=core outcome set; EMA=European Medicines Agency; FDA=US Food and Drug Administration.

## Discussion

### Summary of findings

In this analysis, just under 40% of recent (2015-19) core outcome sets were on topics with relevant EMA or FDA regulatory guidance documents. A median of 70% of outcomes in core outcome sets were specific or general matches to outcomes in EMA guidance documents, and almost half (46%) were specific (ie, exact) matches. Corresponding proportions for pairs of core outcome sets and FDA guidance documents were about half (52%) and quarter (26%), respectively. When outcomes were generally matched, the outcome from the core outcome set was more frequently narrower than the guidance outcome.

### Numbers of outcomes per core outcome set

Our analysis found a generally inverse association between the number of outcomes in a core outcome set and the percentage of those outcomes that were either specific or general matches to outcomes recommended in corresponding guidance documents, both EMA and FDA. This finding contradicts the lack of a relation previously shown by us between the number of outcomes in core outcome sets and the percentage of overlap with outcomes in systematic reviews on the same topic.^9^ We believe that because of the purpose that regulatory guidance documents (examined in the current analysis) serve, they can focus on the most important outcomes of interest for the research question. Core outcome sets with fewer outcomes are also more likely reflect developers’ efforts to prioritise the most important outcomes. These factors might explain why core outcome sets with fewer outcomes in the current analysis had a greater percentage overlap with outcomes in regulatory guidance. It is possible that developers of core outcome sets who recommend a greater number of outcomes adopt a more inclusive view of what a core outcome set should constitute. Another potential reason for the difference between our current and previous findings (from a similar analysis comparing core outcome sets and systematic reviews)^9^ could relate to the different purposes and intended audiences of regulatory guidance documents and systematic reviews.

### Regulatory document purposes

When regulatory bodies, such as the EMA and the FDA, issue guidance on study design (including outcome choice), the guidance is intended to guide the design, analysis, and reporting of studies that evaluate regulated products.^30^ But, regulatory bodies approve claims made in product labels, not the products themselves. So, regulators are often flexible about which outcomes are included in trials because the general focus of regulators is on determining whether the evidence presented supports the claim about clinical benefit that the manufacturer wants included on the product label.

In the context of regulatory document purposes, surrogate (ie, intermediate) outcomes are also worth discussing. Because trials might not be powered for, or might not have long enough follow-up for, longer term clinical outcomes, it is plausible that regulatory guidance for such studies is more accepting of surrogate outcomes than are developers of corresponding core outcome sets (who generally first prioritise outcomes based on importance rather than feasibility). Kalf and colleagues conducted a cross sectional analysis and suggested that although practices vary across regulators, regulators generally accept surrogate outcomes.^31^ However, the issue of surrogate outcomes in recent FDA guidance on emerging disease modifying treatments for dementia^32^ has been a source of concern^33^ and merits careful consideration and clearer methodological guidance. In the current analysis, we did not evaluate the extent to which surrogate outcomes were recommended and how that might vary comparing regulatory documents and core outcome sets. This area has potential for future research.

### Implications for developers of core outcome sets

At the outset, developers should consider the eventual uptake of the core outcome set being developed. Developers should appraise related regulatory guidance documents in the topic area when planning a core outcome set; none of the included sets in the current analysis reported doing this. We also agree with Aiyegbusi and colleagues, who conducted a systematic review of core outcome sets and regulatory guidance and noted that regulators might be relevant stakeholders in outcomes recommended in core outcome sets (especially for those sets related to drugs, devices, and gene therapy); greater collaboration between developers and regulators is warranted; and regulators should participate in the development of core outcome sets.^34^ Regulator participation might be important because it could enable regulators' preferences (along with those of others) to be considered in the development process for core outcome sets. However, bureaucratic restrictions could preclude regulator participation in core outcome sets. How best to engage regulators in the development and adoption process of core outcome sets remains to be explored. Nevertheless, greater adoption of core outcome sets in regulatory guidance documents has the potential to push clinical trialists, especially those funded by industry, to measure and report outcomes from these sets.

### Implications for regulators

The processes of outcome selection for inclusion in regulatory guidance documents generally includes an initial selection by agency staff, followed by input obtained through meetings with clinical experts, patients, and various stakeholders, and engagement with the public.^35^ Although this input might sometimes include alerting regulators about relevant core outcome sets, the extent to which the sets are being consistently considered in this process is unclear.

Regulators are, however, increasingly recognising the importance of engaging patients in the regulatory process.^36 37^ As part of its efforts for Patient-Focused Drug Development, the FDA has developed a set of methodological guidance documents to help various stakeholders "collect and submit patient experience data and other relevant information from patients and caregivers for medical product development and regulatory decision making."^38^ All the core outcome sets examined in the current analysis (and almost 40% of those sets developed by 2019^7^ and over 90% of ongoing sets (unpublished work)) have involved patients in the development process. Regulators should capitalise on this engagement of patients (and various other stakeholders), which is a great strength of the development process for core outcome sets.

Regulators should also be reassured that the overlap in outcomes between core outcome sets and existing corresponding regulatory documents is good. The current analysis finds that as many as 70% of outcomes in core outcome sets were matched to outcomes in corresponding EMA guidance documents and 52% to outcomes in corresponding FDA guidance documents. Thus, core outcome sets could serve as a useful resource for regulators when recommending outcomes for studies evaluating the regulated products.

The majority of core outcome sets in this analysis were published in the same year or after the matched guidance document (66% of COS-EMA pairs and 60% of COS-FDA pairs). Nevertheless, our results support the two main actions that have been suggested for regulators.^12^ Firstly, when drafting a new or updated guidance document, regulators should review the COMET Database for evidence about relevant, high quality, core outcome sets for the scope of the guidance, and consider those outcomes when developing regulatory guidance. Secondly, regulators should engage with the development process of core outcome sets to help identify barriers and facilitators early on.^12^

### Challenges in conducting this analysis

We encountered some challenges during this analysis that are worth discussing. Firstly, we included core outcome sets for research, recognising that these are not only intended for randomised trials but also for non-randomised studies. However, this choice was reasonable because regulatory guidelines issued by the EMA or FDA target both randomised trials and non-randomised studies. Secondly, in some instances, we had to make particularly careful decisions regarding the match in scope between the core outcome set and the guidance document. For example, one eligible core outcome set addressed interventions for patients on haemodialysis. We found an EMA guidance document addressing primary prevention of chronic kidney disease in groups at risk or secondary prevention (ie, early interventions to prevent worsening of kidney function). We did not consider the core outcome set and the guidance document to be a match because haemodialysis is an example of a treatment for established and advanced chronic kidney disease (ie, haemodialysis is a form of tertiary prevention). Thirdly, when extracting outcomes from guidance documents, it was not always clear whether the document was truly recommending a particular outcome. This extraction required judgment in discerning potentially ambiguous language—for example, that the outcome "should," "could," "might," or "may" be considered, or that the outcome "is important." Some of this ambiguity in language could have arisen because guidance from regulatory bodies regarding outcome choices in studies of regulated products generally is non-binding.

### Limitations of the study

The current analysis has some limitations. Firstly, the core outcome sets we analysed were restricted to those with patient involvement in consensus generation, which is what is recommended by COS-STAD.^2^ However, these sets might not be representative of all core outcome sets. Among all core outcome sets published by 2020, those that involved patients included a somewhat larger number of outcomes (median 8, interquartile range 5-12) than those that did not involve patients (6, 3-8; COMET, personal communication). Therefore, given the inverse relation between the number of outcomes in a core outcome set and the percentage overlap between core outcome sets and regulatory outcomes (documented in this paper), the overlap may have been higher if we looked at a sample of core outcome sets without patient involvement. The core outcome sets in the current analysis represented a recent five-year sample, and the level of adherence to COS-STAD standards (including patient involvement) has generally been improving.^7 24–27^

Secondly, although we applied our systematic search methods for guidance documents consistently, some guidance documents from the EMA or FDA could have been missed. Thirdly, for regulatory guidance documents, we restricted our search to the EMA and FDA. Although the EMA and FDA are two prominent healthcare regulatory bodies, we recognise that there are other regulatory bodies in other regions and countries. As such, our findings should be inferred to be informed by guidance produced by the EMA and FDA.

### Conclusions

We found sizeable overlap between outcomes in core outcomes sets and in corresponding EMA and FDA guidance documents. We encourage developers to involve regulators in core outcome set development and to consider outcomes recommended in regulatory guidance documents. We encourage regulators to engage with the development process of core outcome sets (to help identify barriers and facilitators early on) and, when drafting a new or updated guidance document, to review the COMET Database for relevant high quality core outcome sets.

## Data Availability

Data are available upon reasonable request.
