# Supplementary figures and images for: Comparison of published core outcome sets with outcomes recommended in regulatory guidance from the US Food and Drug Administration and European Medicines Agency: cross sectional analysis
Source: BMJ Med. 2022 Nov 3;1(1):e000233. doi: 10.1136/bmjmed-2022-000233 (PMC9978677; doi:10.1136/bmjmed-2022-000233)

## A) COS and EMA

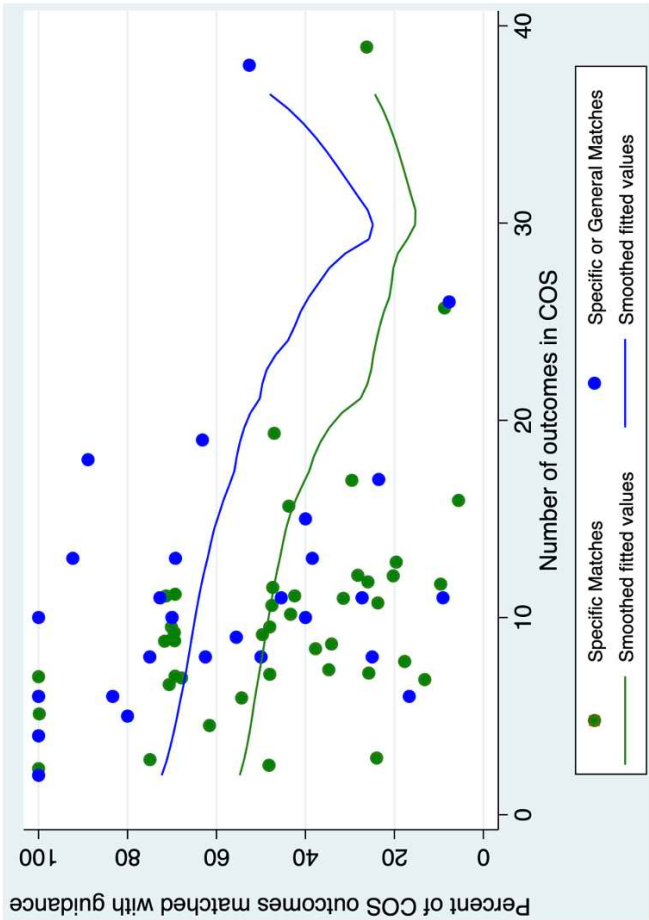

## B) COS and FDA

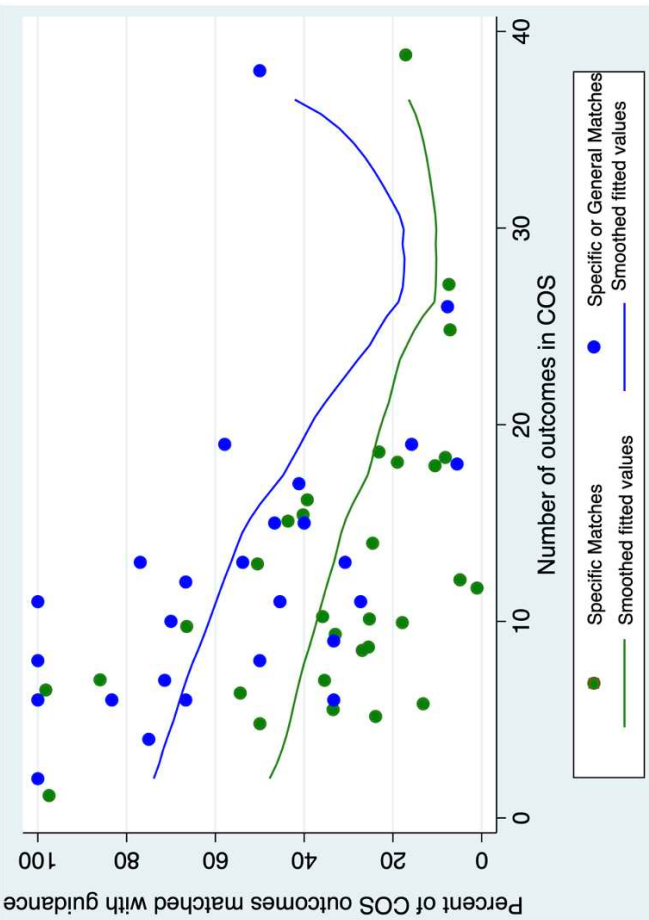

Supplement: Supplementary data [file bmjmed-2022-000233supp001.pdf]
